# Rational design of red AIEgens with a new core structure from non-emissive heteroaromatics†
†Electronic supplementary information (ESI) available: Materials and methods, synthetic procedures, biological experiment, structural characterization, crystallographic date, cyclic voltammetry curves, computational details, UV-vis and PL spectra, SEM and TEM image characterization of nanoparticles and cell imaging. CCDC 1822148 and 1822244. For ESI and crystallographic data in CIF or other electronic format see DOI: 10.1039/c8sc02810a


**DOI:** 10.1039/c8sc02810a

**Published:** 2018-08-21

**Authors:** Ming Chen, Xianglong Hu, Junkai Liu, Baixue Li, Nelson L. C. Leung, Lucia Viglianti, Tsz Shing Cheung, Herman H. Y. Sung, Ryan T. K. Kwok, Ian D. Williams, Anjun Qin, Jacky W. Y. Lam, Ben Zhong Tang

**Affiliations:** a Department of Chemistry , Hong Kong Branch of Chinese National Engineering , Research Center for Tissue Restoration and Reconstruction , Institute of Advanced Study , State Key Laboratory of Molecular Nanoscience , Division of Life Science and Diversion of Biomedical Engineering , The Hong Kong University of Science and Technology , Clear Water Bay, Kowloon , Hong Kong , China . Email: tangbenz@ust.hk; b NFSC Center for Luminescence from Molecular Aggregates , SCUT-HKUST Joint Research Institute , State Key Laboratory of Luminescent Materials and Devices , South China University of Technology , Guangzhou 510640 , China; c MOE Key Laboratory of Laser Life Science , Institute of Laser Life Science , College of Biophotonics , South China Normal University , Guangzhou , 510631 , China; d HKUST-Shenzhen Research Institute , Shenzhen 518057 , China

## Abstract

The development of new aggregation-induced emission (AIE) systems is a hot research topic, from which functional materials with diversified structures and properties are derived. Here, based on rare, non-emissive and highly electron-withdrawing heteroaromatics of 1,4,5,8-tetraazaanthracene (TAA), experimental and theoretical studies reveal that attaching phenyl rotors to TAA is crucial to creating a new N-type AIE core structure. Furthermore, by covalent attachment of electron-donating aromatic amines to the peripheries of the AIE core, red AIEgens could be obtained readily, which exhibit excellent photostability for long-term lysosome tracking. This work not only provides a new strategy to design heterocycle-containing AIEgens from non-emissive heteroaromatics but also stimulates more their applications as bio-imaging materials.

## Abstract 2

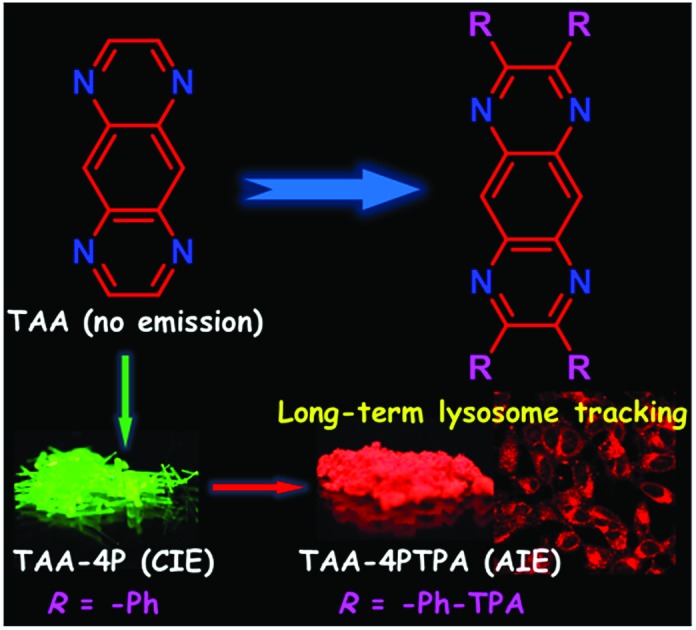
This work provides a new strategy to design heterocycle-containing AIEgens from non-emissive heteroaromatics and promotes their applications for long-term lysosome imaging.

## 1. Introduction

The exploitation of aggregation-induced emission luminogens (AIEgens) is both of great academic and practical importance and can stimulate rapid development in the areas of optoelectronics, bio-imaging, nanoscience, *etc.*^1^,^2^ To date, hundreds of AIEgens have been developed. However, they are often derived from the same core structure. For example, tetraphenylethene (TPE) is one of the widely used luminogenic cores and the majority of AIE research is focused on TPE derivatives because they are easy to be synthesized and post-modified.^3^–^5^ On the other hand, recent studies have shown that the double bond in its structure plays an important role in the AIE working mechanism.^6^–^8^ Thus, much debate remains in the mechanistic understanding. Additionally, photo-oxidation and photo-activation are apt to occur in TPE systems to deteriorate their stability.^9^–^12^ Such obstacles are also encountered in other AIEgens with double bonds in their core structures, such as distyrylanthracene, tetraphenyl-1,4-butadiene, triphenylethene, *etc.*^13^–^15^

The AIE phenomenon of several heterocycle-based AIE systems, such as phenyl-substituted siloles, pyrazines, pyrroles and oxazoliums, can be well explained by the restriction of the intramolecular motion (RIM) mechanism because of the absence of independent double bonds in their structures.^16^–^23^ Meanwhile, their stability is pretty high by virtue of their whole aromatic conjugated structure. However, most of them are difficult to synthesize and purify. Moreover, their emissions are mainly centered in the blue light region, which greatly hampers their practical applications, especially in biological imaging. This makes the development of new heterocycle-based AIEgens very promising but challenging.

**Chart 1:**
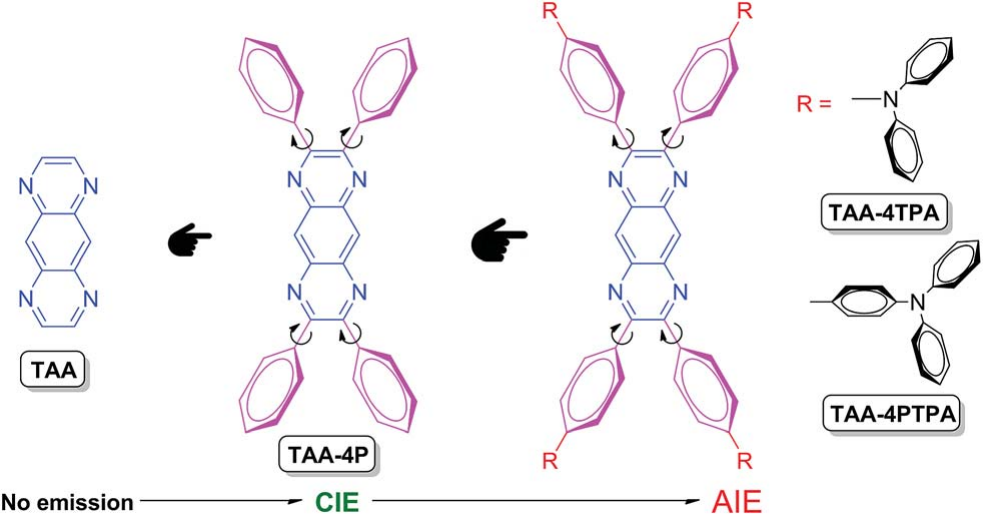
Structure and property evolution from TAA to TAA-containing luminogens.

AIE research has been ongoing for more than 10 years but it is still a young area of research in comparison with conventional aggregation-caused quenching (ACQ) systems. In view of a large number of ACQ luminophores, how to change their emission behavior to AIE is very attractive. To achieve such a target, we had chemically decorated TPE onto ACQ luminophores, like perylene, pyrene and perylene bisimide, and succeeded in generating new AIEgens with high emission efficiency in the solid state.^24^,^25^ The introduction of AIE rotors facilitates the molecular motion in solution, while the π–π stacking was prevented in the aggregated state due to the twisted molecular geometry. On the other hand, many heteroaromatics are well conjugated but are not emissive in any state.^26^,^27^ For example, 1,4,5,8-tetraazaanthracene (TAA) can be regarded as a nitrogen-substituted derivative of anthracene (Chart 1, Scheme S1 and Fig. S1–S3†). The presence of nitrogen atoms in the molecular structure significantly imparts TAA with good electron affinity (Fig. S4†). Indeed, Bunz and Miao designed and synthesized analogues of TAA, which showed a high electron mobility larger than 10 cm^2^ V^–1^ s^–1^ when functioned as an N-channel semiconductor in an organic thin-film transistor.^28^–^31^ The utilization of TAA as a luminescent material, however, is impeded because its inherent n → π* transition has efficiently quenched the light emission (Fig. S5 and S6†). Therefore, it is particularly interesting to utilize TAA as a building block to create luminescent materials with novel structures and to provide new clues for preparing heteroaromatics-containing AIEgens. Besides, because TAA shows strong electron-withdrawing ability, it can help to construct a novel N-type AIE core to build red-emissive AIEgens *via* rational structural design.

In this work, we presented our effort in utilizing TAA for AIEgen fabrication. Detailed investigation suggests that: (1) the modification of TAA with phenyl rotors will decrease the component of the n → π* transition but in turn increase the component of the π → π* transition to open up the radiative transition channel of derivatives and (2) the flexible rotors introduced into TAA can tune the luminescence behavior of its derivatives and endow them with AIE features. According to this rule, a new N-type AIEgen was generated in one step reaction based on TAA. It exhibited green emission in the crystal state and strong electron-withdrawing ability. Using this TAA derivative as an AIE core and decorating its peripheries with strong electron-donating aromatic amines provides AIEgens readily with red emissions. The fabricated nanoparticles with red AIEgens display excellent stability for long-term lysosome imaging. This work thus plots a new strategy for designing AIEgens from non-emissive heteroaromatics and stimulates more luminescence applications for heterocycle-containing compounds.

## 2. Results and discussion

The derivatives of TAA, named TAA-4P, TAA-4TPA and TAA-4PTPA, were prepared *via* facile steps under mild conditions according to the literature (Chart 1 and Scheme S2†).^32^–^35^ All products were characterized by standard spectroscopy methods with satisfactory data corresponding to their structures (Fig. S7–S16†).

**Fig. 1:**
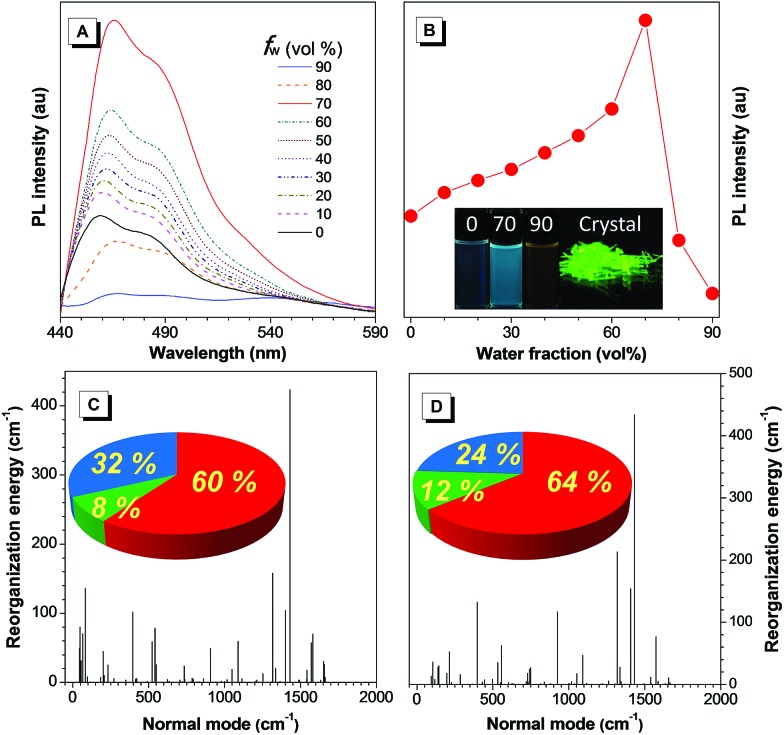
(A) PL spectra of TAA-4P in THF/water mixtures containing different water fractions (*f*_w_) [TAA-4P] = 10 μM, *λ*_ex_ = 428 nm. (B) Plot of PL intensity *versus* the composition of the THF/water mixtures of TAA-4P. Inset: photographs of TAA-4P in THF/water mixtures with water fractions of 0, 70% and 90%, and in the crystal state. (C and D) Reorganization energy *versus* normal mode wavenumbers of TAA-4P in (A) THF solution and (B) the crystal state. Inset: projection of reorganization energy on the internal coordination in (C) THF solution and (D) the crystal state (red, green and blue stand for the bond length, bond angle and dihedral angle, respectively).

Since TAA is non-emissive, how to transform it to be luminescent or even AIE-active through molecular engineering is interesting and challenging. According to our previous research, the decoration of flexible phenyl rotators onto a benzene core is crucial for tuning the molecular luminescence behavior.^36^ We thus designed TAA-4P with TAA modified with four phenyl rings in the periphery. PL spectra of TAA-4P in THF/water mixtures were taken to study its properties. Unlike TAA, TAA-4P in THF gave weak sky-blue emission with peaks located at 458 and 478 nm. After the addition of water, the emission enhanced gradually until the water fraction (*f*_w_) reached 70% with the shape of the emission peak less changed. Afterwards, remarkably quenching occurred when *f*_w_ increased 80% and 90%, and nearly no emission of TAA-4P could be observed when *f*_w_ = 90% (Fig. 1A and B). Because TAA-4P is hydrophobic, THF and water act as good and poor solvents, respectively. The morphology of TAA-4P was correlated with the mixed solvent with various ratios, which further determined the different luminescence behavior. For further examination, dynamic light scattering (DLS) analysis for the dispersion of TAA-4P in different mixed solvents was carried out. In pure THF, TAA-4P was molecularly dissolved, giving weak emission. After the addition of water, nanoaggregates started to form when *f*_w_ = 40%. The luminescence intensity was basically enhanced as the aggregate size increased from 81 nm to 373 nm when the *f*_w_ changed from 40% to 70%. However, further increasing the *f*_w_ decreased the size of the aggregate (170 nm and 99 nm for 80% and 90%, respectively), where the emission was accordingly quenched (Fig. S17†).

Because the strongest and most quenched emissions were related to aggregates formed in the THF/water mixtures with an *f*_w_ of 70% and 90%, respectively, a scanning electron microscope (SEM) was utilized to further examine the aggregate morphology after solvent evaporation (Fig. S18A and C†). In the former case, TAA-4P assembled into very ordered rods with a length and width of *ca.* 40 and 1 μm, respectively. Powder X-ray diffraction (PXRD) measurement of the rod sample showed sharp diffraction peaks in the diffractogram, indicating that they are crystalline (Fig. S19†). As the size of the rods was much larger than that of the nanoaggregates (280 nm) obtained by DLS, it is concluded that TAA-4P first formed smaller crystalline aggregates in the THF/water mixture and then further assembled into bigger rods when the solvent was evaporated. Thus, the enhanced emission in the THF/water mixture (*f*_w_ = 70%) compared to pure THF solution was attributed to the formation of crystalline aggregates. In comparison, at *f*_w_ = 90%, few rod-like assemblies were observed, but many disordered aggregates with a much smaller size were found with a diameter of *ca.* 150 nm, which was very close to the result (99 nm) from DLS analysis. It was mostly due to the generation of amorphous aggregates in the mixed solvent, which made it difficult to undergo further assembly. Similar results were also observed by using a transmission electron microscope (TEM) (Fig. S18B and D†). Therefore, TAA-4P was an AIEgen with crystallization-induced emission (CIE) properties. The absolute quantum yields (*Φ*_F_) of TAA-4P in THF and crystals were recorded as 1.9% and 3.5% by integrating spheres, which also affirmed this conclusion (Table S1†). Moreover, cyclic voltammetry study revealed that the LUMO (LUMO = lowest unoccupied molecular orbital) energy level of TAA-4P was just as low as that of TAA, demonstrating that it was a typical N-type AIEgen (Fig. S4†).

Theoretical simulation based on the (TD) B3LYP/6-31G(d) level in tetrahydrofuran (THF) and in the crystal state modeled by PCM and ONIOM approaches gave the result that a remarkably enhanced oscillator strength (*f*) of the S_1_ → S_0_ transition of TAA-4P was obtained as 0.33 and 0.19 in solution and the crystal state, in which the transition between the LUMO and HOMO (HOMO = highest occupied molecular orbital) accounted for 94% and 89%, respectively (Fig. S20†). Based on the optimized geometry in the S_1_ state of TAA-4P, the LUMO and HOMO were found to demonstrate π character, indicating that the allowed π → π* transition dominated the emission of TAA-4P. Thus, the emission of the TAA derivative was lit up with the enhanced radiative decay channels as the transition component changed from the n → π* transition to the π → π* transition due to the introduction of phenyl rotors. The crystal structure of TAA-4P indicated that the attached phenyl rings twisted against the central plane with an average dihedral angle of 44° (Fig. S21†). However, how did these phenyl rotors influence the AIE behavior of TPP-4P? To gain a deeper insight into the process, the reorganization energy (*λ*) of TAA-4P in THF and the crystal state was investigated, since it is one of the key factors to affect the non-radiative transition. The total *λ* in THF and the crystal state was calculated to be 1900 and 1704 cm^–1^, respectively, using the MOMAP package,^36^–^40^ indicating that non-radiative decay was suppressed from the solution to the crystal state. From the plots of *λ versus* normal frequencies of TAA-4P (Fig. 1C and D), the low frequency vibration modes assigned to the rotation of peripheral phenyl rings were reduced significantly in the crystal state compared to the solution state, whereas higher frequency vibration modes corresponding to the stretching and bending motion of bonds saw less changes. Furthermore, casting the *λ* onto the internal coordinate depicted that variation in both the bond length and the dihedral angle contributed obviously to the total *λ*. Since the bond-stretching vibration only takes places with a distance of *ca.* 1 Å along the bond axis, which is relatively insensitive to the aggregation environment, the contribution from the changes of the dihedral angle decreased from 32% to 24% upon aggregation into the crystal state, which was thus considered as the main contributor affecting non-radiative decay channels between the two states. All these results indicated that the restriction of rotation of the phenyl ring of TAA-4P played an important role in determining the CIE effect.

Nevertheless, the restriction in energy transfer cannot account for the AIE mechanism here, because TAA-4P packed in a parallel fashion as observed from molecular packing in single crystals.^41^ Meanwhile, it was found that there is some intermolecular interaction to stabilize the packing because of the presence of overlap of π electron clouds between adjacent molecules (Fig. S22†).^42^,^43^

**Fig. 2:**
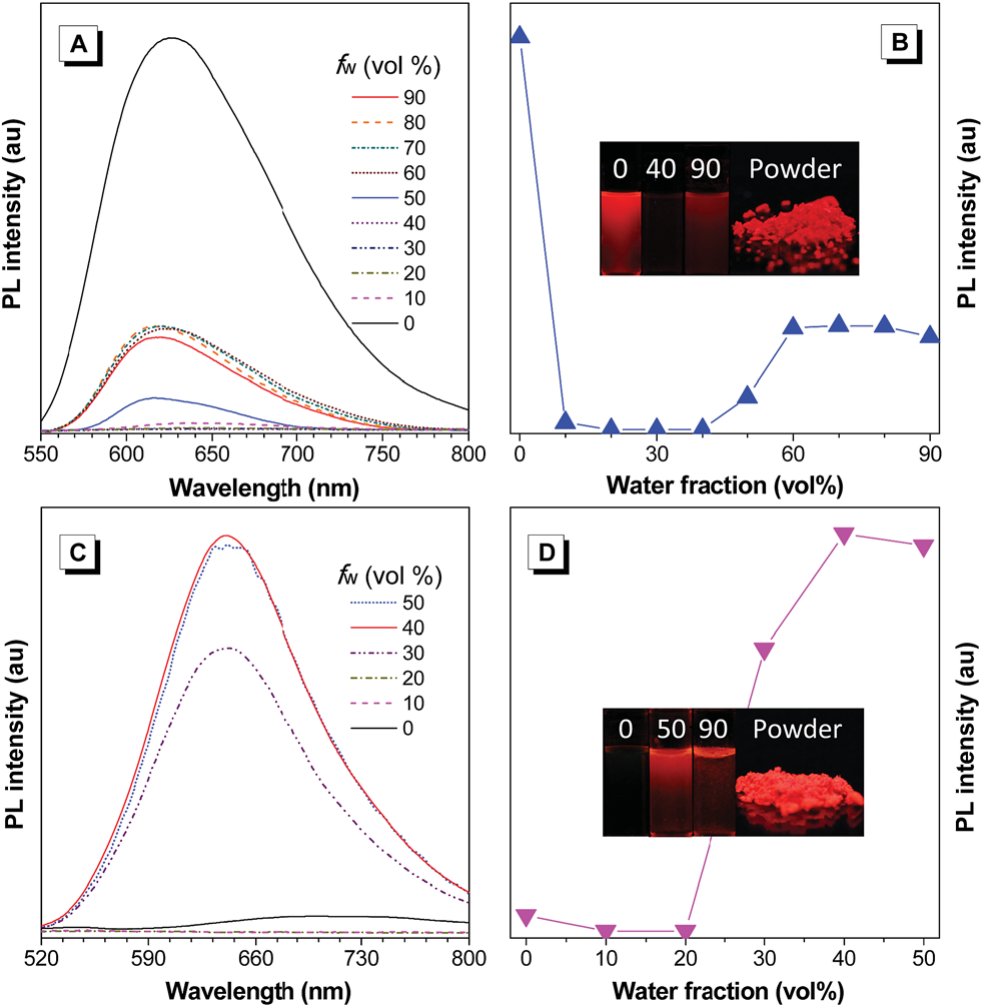
(A and C) PL spectra of (A) TAA-4TPA and (C) TAA-4PTPA in THF/water mixtures containing different water fractions (*f*_w_), [TAA-4TPA] = [TAA-4PTPA] = 10 μM, and *λ*_ex_ for TAA-4TPA and TAA-4PTPA is 520 nm and 474 nm, respectively. (B and D) Plot of PL intensity *versus* the composition of the THF/water mixtures of (B) TAA-4TPA and (D) TAA-4PTPA. Inset in (B and D): photographs of (B) TAA-4TPA in THF/water mixtures with water fractions of 0, 40% and 90%, and in the powder state and (D) TAA-4PTPA in THF/water mixtures with water fractions of 0, 50% and 90%, and in the powder state.

Based on the new AIE-active core structure TAA-4P and its strong electron affinity, we modified it with four strong electron-donating diphenylamines to generate TAA-4TPA. Because of the involved D–A effect, TAA-4TPA would likely be AIE-active with significantly red-shifted emission. To check it, we also studied the PL spectra of TAA-4TPA in THF/water mixtures containing different *f*_w_ (Fig. 2A and B). In THF, TAA-4TPA displayed strong red emission at 627 nm. However, after the addition of water with an *f*_w_ of 10%, the emission was almost quenched, while such behavior was kept until the *f*_w_ reached 40%. Then, an obvious emission centered at 620 nm rose when the *f*_w_ was further increased. As water is much more polar than THF, the addition of water to THF will increase the polarity of the mixed solvent, thus quenching the emission of molecules by the solvent effect. After the addition of a large amount of water (*f*_w_ ≥ 50%), aggregation would occur where the RIM was activated to show AIE. However, the emission intensity of TAA-4TPA in aggregates was still much weaker than that in THF, making it possible to further rationally optimize its molecular structure to tune the properties.

We supposed that attaching additional rotatable phenyl rings between TAA-4P and diphenylamine moieties would promote the molecular motion to quench emission in the solution while keeping the AIE activity. We thus synthesized the molecule of TAA-4PTPA with the desired structure. As expected, TAA-4PTPA exhibited faint but much redder emission at *ca.* 708 nm in THF. The emission was further quenched with a subtle increase of *f*_w_. A strong emission band at 641 nm was recorded with *f*_w_ started from 30%. Because of the additional conjugated units, the solubility of TAA-4PTPA in THF was decreased, making it aggregate at lower *f*_w_, in comparison to TAA-4TPA. Moreover, at high *f*_w_ from 60% to 90%, macroscopic aggregates were formed immediately and floated on the surface of the solvent upon water addition. Thus, their spectra were hard to detect by using a fluorescence spectrometer (Fig. 2C and D).

**Fig. 3:**
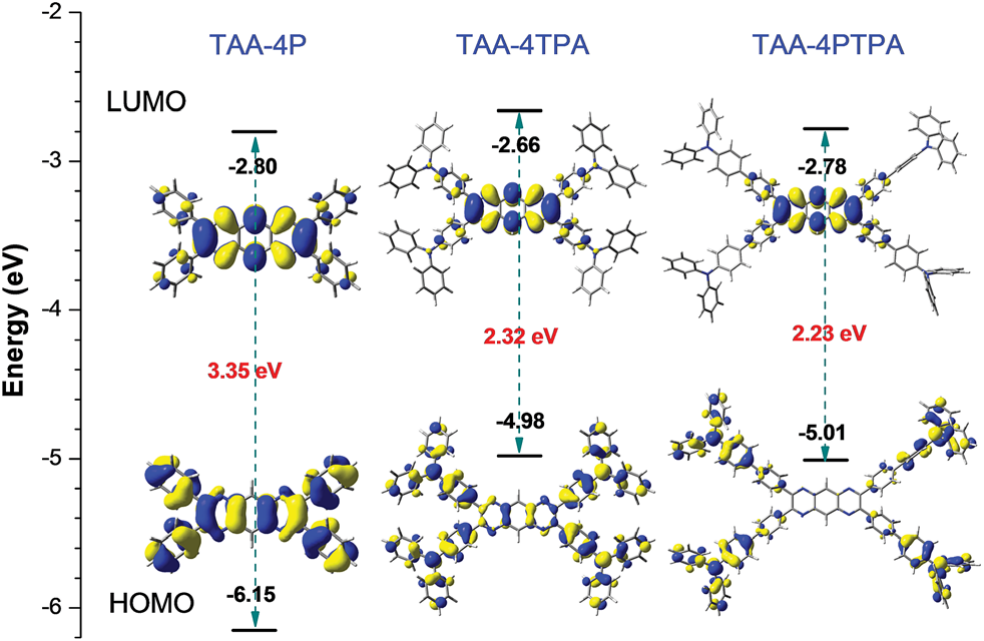
Energy levels and electron cloud distribution of the HOMO and LUMO of TPP-4P, TAA-4TPA and TAA-4PTPA calculated at the B3LYP/6-31G(d) level in THF modeled by the PCM approach.

Similar to TAA-4P, both TAA-4TPA and TAA-4PTPA adopted twisted S_0_ geometry as optimized at the B3LYP/6-31G(d) level in THF (Fig. 3 and Table S3†). The calculation also revealed that the HOMO is distributed on the whole molecule and mainly centered at the triphenylamine units of TAA-4TPA and TAA-4PTPA, respectively, while the LUMO is located on the TAA-4P part of both molecules. It was obvious that TAA-4TPA possessed good molecular conjugation because its electron cloud was extensively delocalized on the whole molecule. Thus, its structure was much rigid. It also indicated that the transition between the HOMO and LUMO of TAA-4TPA was composed of mixed properties of a local excited (LE) transition and charge transfer (CT) transition, whereas, in TAA-4PTPA, the CT transition became dominant. For ease of understanding of the photo-physical process of the two molecules, the proposed energy level diagrams were given (Fig. S23†). When TAA-4TPA was excited, no matter which excited state the electron would reach, it would relax quickly to the lowest vibration state of S_1_ by internal conversion or vibrational relaxation. Because TAA-4TPA was rigid, a less molecular conformational change occurred during the relaxation process, leading to mixed LE and CT emission from S_1_ to S_0_. However, the flexible molecular conformation of TAA-4PTPA allowed it to relax to a twisted intramolecular charge transfer (TICT) state easily by molecular motion upon excitation, which thereby induced much redder emission. It should be noted that the HOMO of TAA-4PTPA calculated by using the (TD) B3LYP/6-31G(d) level was very different from that based on S_0_ geometry, indicative of a large conformation difference between the two vibration states, which allowed for remarkable relaxation (Table S4†). The Stokes shift of TAA-4PTPA (234 nm) was much larger than that of TAA-4TPA (107 nm), also confirming that powerful molecular motion was apt to take place in TAA-4PTPA to dissipate energy after excitation (Table S1†). The excited-state decay rates were estimated by using formulae of *k*_r_ = *Φ*_F_/*τ* and *k*_nr_ = (1 – *Φ*_F_)/*τ*, where *k*_r_ and *k*_nr_ were the radiative and non-radiative decay rates, respectively. The calculated *k*_nr_ of TAA-4TPA in THF was 2.49 × 10^8^ s^–1^, which was lower than that of TAA-4PTPA (6.23 × 10^8^ s^–1^). On the other hand, the *k*_r_ of the former (0.61 × 10^8^ s^–1^) was much higher than that of the latter (0.06 × 10^8^ s^–1^), which was also due to its stronger molecular rigidity, giving rise to a less conformational change under relaxation but a larger overlap between the HOMO and LUMO. Both factors enabled TAA-4TPA to give a much higher *Φ*_F_ (19.7%) than TAA-4PTPA (1%) in solution. However, in the solid state, the motion of TAA-4PTPA was better restricted. Meanwhile, its radiative transition between the LUMO and HOMO was less affected by small structural relaxation due to the steric constraint. Therefore, TAA-4PTPA showed a higher *Φ*_F_ of 8.0% than TAA-4TPA in the solid state (Table S1†).

**Fig. 4:**
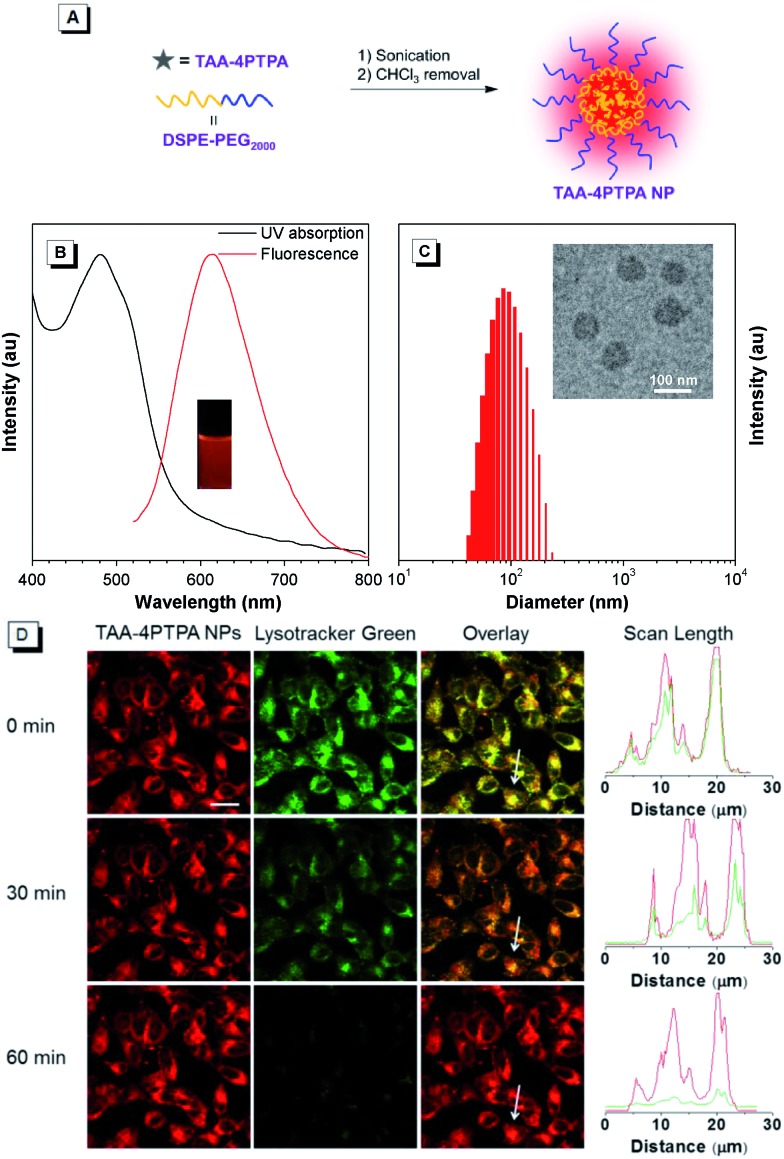
(A) Schematic illustration of the fabrication of TAA-4PTPA NPs with TAA-4PTPA and DSPE-PEG_2000_. (B) UV-vis and PL spectra of TAA-4PTPA NPs in water. Inset: photograph of TAA-4PTPA NPs taken under irradiation of 365 nm UV light. (C) Particle size distributions of TAA-4PTPA NPs studied by DLS. Inset: their TEM image. (D) CLSM images of HeLa cells after incubation with TAA-4PTPA NPs after *in situ* continuous 488 nm laser irradiation on a CLSM imaging system for different durations, and the dye concentration is 2 μM. The lysosomes were stained with Lysotracker Green, the excitation wavelength was 488 nm, and the emission channel was set to be 500–560 nm and 600–730 nm for Lysotracker Green and TAA-4PTPA NPs, respectively. The scale bar is 20 μm.

Finally, as a proof of concept, we explored the biological application of red-emissive TAA-4PTPA for bio-imaging due to its low photo-damage to living cells and small overlap with bio-substrate autofluorescence.^44^–^46^ TAA-4PTPA was hydrophobic, and thus amphiphilic phospholipid, 1,2-distearoyl-*sn*-glycero-3-phosphoethanolamine-*N*-[methoxy(polyethylene glycol)-2000] (DSPE-PEG_2000_) was used to encapsulate the molecules and formulate nanoparticles (TAA-4PTPA NPs) with good water stability (Fig. 4A).^47^,^48^ The NPs showed obvious red emission peaked at 615 nm as a result of the AIE activity of TAA-4PTPA (Fig. 4B). The TEM image indicated that the NPs had a spherical shape with a diameter of *ca.* 75 nm, which was somewhat smaller than the result obtained from DLS analysis (*ca.* 96 nm) due to the invisible hydrophilic coronas under TEM observation (Fig. 4C). The *in vitro* cytotoxicity of the NPs against HeLa cells was evaluated by 3-(4,5-dimethylthiazol-2-yl)-2,5-diphenyltetrazolium bromide (MTT) assay. The cells showed more than 85% viability after incubation with the NPs for 24 h at dye concentrations ranging from 0.14 to 20 μM, indicative of minimal toxicity for bio-application (Fig. S25†).

After that, red-emissive TAA-4PTPA NPs were assessed for cell imaging. HeLa cells were incubated with the NPs and imaged by using a Confocal Laser Scanning Microscope (CLSM) under 488 nm laser excitation, and the emission signals were collected at 600–730 nm. A remarkable overlap between the fluorescent pixels of TAA-4PTPA NPs and commercial Lysotracker Green indicated the lysosome-specific imaging potency for the NPs (Fig. 4D). We then evaluated the long-term photo-stability of the NPs, because it is one of the crucial parameters for developing fluorescent imaging agents. The examination of persistent photo-stability was performed in parallel for the NPs and commercial Lysotracker Green by continuous 488 nm laser irradiation for 1 h during real-time cell imaging. As shown in Fig. 4D, the NPs displayed a strong signal in lysosome imaging from the start, but a less signal loss (<10%) occurred when the irradiation time extended to 1 h. However, the signal of Lysotracker Green was significantly reduced even when irradiated for only 0.5 h and almost lost at 1 h. Besides, the *in vitro* stability of the NPs under acidic conditions was examined at pH = 5, which simulated the acidic environment of lysosomes. A minimal change was observed in the PL spectra of TAA-4PTPA NPs even upon incubation for one week at pH = 5, suggesting excellent stability in the harsh lysosomal acidic medium (Fig. S26†). Collectively, TAA-4PTPA based nanoparticles are very promising for long-term lysosome imaging and tracking.

## 3. Conclusions

In this work, based on the non-emissive and highly electron-withdrawing heteroaromatics of TAA. Our study proved that: (1) attaching TAA with phenyl rotors can change the transition component from the n → π* transition to the π → π* transition, thus increasing the irradiative transition component of TAA derivatives and (2) the introduction of phenyl rotors into TAA facilitated the rotation of molecules in solution to dissipate energy after excitation non-radiatively, while the motion was suppressed to open up the irradiative transition channel upon aggregation, endowing the derivatives with AIE activity. By utilizing this rule, an N-type AIE core structure of TAA-4P was designed and further decoration of TAA-4P with electron-donating aromatic amines created red AIEgens readily, which can be fabricated into nanoparticles for long-term lysosome imaging. This work not only provides a strategy for designing heteroaromatics-containing AIEgens, but also stimulates more enthusiasm for developing functional materials based on heteroaromatic compounds.

## Conflicts of interest

There are no conflicts to declare.

## Supplementary Material

Supplementary information

Crystal structure data
