# Supplementary material for: Rational design of red AIEgens with a new core structure from non-emissive heteroaromatics
Source: Chem Sci. 2018 Aug 21;9(40):7829–34. doi: 10.1039/c8sc02810a (PMC6194487; doi:10.1039/c8sc02810a)
Supplement: Supplementary file 1 [file SC-009-C8SC02810A-s001.pdf]

Electronic Supplementary Material (ESI) for Chemical Science.
This journal is © The Royal Society of Chemistry 2018

Supporting Information

# Rational Design of Red AIEgens with New Core Structure from Non-Emissive Heteroaromatics

Ming Chen,‡[a] Xianglong Hu,‡[a,c] Junkai Liu,‡[b] Baixue Li,[b] Nelson L. C. Leung,[a] Lucia Viglianti,[a] Tsz Shing Cheung,[a] Herman H. Y. Sung,[a] Ryan T. K. Kwok,[a] Ian D. Williams,[a] Anjun Qin,[b] Jacky W. Y. Lam[a] and Ben Zhong Tang*[a,b,d]

[a] Department of Chemistry, Hong Kong Branch of Chinese National Engineering, Research Center for Tissue Restoration and Reconstruction, Institute of Advanced Study, State Key Laboratory of Molecular Nanoscience, Division of Life Science and Division of Biomedical Engineering, The Hong Kong University of Science and Technology, Clear Water Bay, Kowloon, Hong Kong, China

[b] NFSC Center for Luminescence from Molecular Aggregates, SCUT-HKUST Joint Research Institute, State Key Laboratory of Luminescent Materials and Devices, South China University of Technology, Guangzhou 510640, China

[c] MOE Key Laboratory of Laser Life Science & Institute of Laser Life Science, College of Biophotonics, South China Normal University, Guangzhou, 510631, China

[d] HKUST-Shenzhen Research Institute, Shenzhen 518057, China.

E-mail: tangbenz@ust.hk

**Experimental Section**

**Materials and Instrumentation**: All chemicals were purchased from Sigma-Aldrich, J&K, Beijing HWRK Chem Co., LTD and used directly without further purification. 1,2-bis(4'- (diphenylamino)-[1,1'-biphenyl]-4-yl)ethane-1,2-dione was prepared according to our previous literature.[1] $^{1}H$ and $^{13}C$ spectra were recorded on a Bruker AVIII 400 MHz NMR spectrometer with $CDCl_3$, $CD_2Cl_3$ or *d*-THF as solvent. High-resolution mass spectra (HRMS) were measured with a GCT premier CAB048 mass spectrometer operated in MALDI-TOF mode. UV-vis spectra were measured using a Cary 50 Conc spectrophotometer. PL spectra were recorded on a Perkin-Elmer LS 55 spectrofluorometer. The absolute quantum yield ($\Phi_F$) was recorded with a Hamamatsu Quantaurus-QY C11347 spectrometer. The PL decay curve was performed on a Hamamatsu Quantaurus-lifetime C11367-11. The size of aggregates was measured by dynamic light scattering of a Brookhaven Zeta potential analyzer. The scanning electron microscope images were taken on a JEOL-6390 scanning electron microscopy. The transmission electron microscopy images were taken with a JEM 2010 transmission electron microscope. The powder X-ray diffraction was carried out with a Philips PW 1830 X-ray Diffractometer. Single-crystal X-ray diffraction was carried out at 100 K on a Bruker–Nonices Smart Apex CCD diffractometer with graphite monochromated Mo Kα radiation. Cyclic voltammetry was performed on a three-electrode cell using CHI610E electrochemical workstation at room temperature. The experiment was carried out in anhydrous dichloromethane with Pt disk, Pt wire and SCE as working, counter and reference electrodes, respectively. N-tetrabutylammonium hexafluorophosphate and ferrocene were used as supporting electrolyte and standard, respectively. The measurement was conducted after the nitrogen was purged with a scanning rate of 50 mV/s.

**Details for calculation:** Geometrical optimizations were carried out at the (TD) B3LYP/6-31G(d) level at the $S_0$ ($S_1$) state for all the studied compounds in the solution phase using Gaussian 09 package. Linear-response (LR) polarizable continuum model (PCM) with $\varepsilon = 7.43$ were adopted to evaluate the real surrounding of tetrahydrofuran solution. Further frequency calculations and state-specific (SS)

PCM were applied for TAA-4P to calculate the excitation energy at the minima of $S_0$ and $S_1$ state. The corresponding calculations for TAA and TAA-4P in the crystal phase were performed using combined quantum chemistry and molecular chemistry (QM/MM) approach in the ONIOM model. The QM/MM model was set up by cutting a cluster from the single crystal structure, the central molecule was treated as the QM part at the (TD) B3LYP/6-31G(d) level and the surrounding ones acted as the MM part with the universal force field (UFF). The reorganization energy in the solution and crystal phase was calculated in the MOMAP package. Considering the higher consistency between values calculated by B3LYP function and experimental data of absorption and emission for the studied compounds, we applied B3LYP function to perform all the calculations. Comparison of calculated values based on B3LYP and long-range correction function CAM-B3LYP and experimental data were listed in the Table S2. The results showed that B3LYP was superior to CAM-B3LYP chosen for theoretical calculation because of much matched calculated spectra corresponding to the experimental data.

**Synthesis of TAA:** The product was prepared according to the literature procedure.[2] Into a 50 mL round bottom flask was added 212 mg (0.75 mmol) of 1,2,4,5,-tetraaminobenzene tetra hydrochloride (**1**), 218 mg (1.5 mmol) of aqueous glyoxal solution (40 wt.%), 246 mg (3 mmol) of sodium acetate and 20 mL of ethanol. The mixture was stirred under reflux for 30 min. Afterwards, the mixture was concentrated by reduce pressure and extracted by dichloromethane. The organic phase was washed by water several times and concentrated by reduce pressure. The crude product was purified on a silica-gel column using dichloromethane as eluent. A pale-yellow powder of 14 mg (0.08 mmol) was obtained in yield of 10%. $^1$H NMR (400 MHz, $CDCl_3$), $\delta$ (ppm): 9.04 (s, 4H), 9.02 (s, 2H). $^{13}$C NMR (100 MHz, $CDCl_3$), $\delta$ (ppm): 147.1, 141.5, 130.2. HRMS (MALDI-TOF): m/z 183.0684 ($[M+1]^+$), calcd for 183.0671). Crystal structure (CCDC 1822148).

**Synthesis of 1,2-bis(4-(diphenylamino)phenyl)ethane-1,2-dione (5):** The product was prepared according to the literature procedure.[3] Into a 50 mL round bottom flask was added 1.29 g (10 mmol) of oxalyl chloride (**4**), 2. 67 g (20 mmol) of anhydrous

aluminium chloride and 50 mL of dichloromethane. Then, 5.84 g (24 mmol) of triphenylamine (**3**) was added in portions. The mixture was stirred under reflux for 6 h. After cooled to the room temperature, the mixture was poured onto the ice water and extracted by dichloromethane. The organic phase was washed by water several times and concentrated by reduce pressure. The crude product was purified on a silica-gel column using hexane/dichloromethane (v/v = 20:1) as eluent. A yellow powder of 1.68 g (3.1 mmol) was obtained in yield of 31%. $^1$H NMR (400 MHz, $CDCl_3$), $\delta$ (ppm): 7.79 (d, $J$ = 8.0 Hz, 4H), 7.35 (t, $J$ = 8.0 Hz, 8H), 7.18–7.15 (m, 12H), 6.96 (d, $J$ = 8.0 Hz, 4H). $^{13}$C NMR (100 MHz, $CDCl_3$), $\delta$ (ppm): 192.7, 152.8, 145.3, 131.0, 129.1, 125.8, 124.7, 124.6, 118.4.

**Synthesis of TAA-4P:** The product was prepared similar to the literature procedure.[4] Into a 50 mL round bottom flask was added 148 mg (0.71 mmol) of benzil, 100 mg (0.35 mmol) of 1,2,4,5,-tetraaminobenzene tetra hydrochloride (**1**) and 20 mL of acetic acid. The mixture was stirred under reflux overnight. After cooled to the room temperature, the crude product was collected by filtration and further purified by recrystallization in acetic acid. A yellow needle-like crystal of 127 mg (0.26 mmol) was obtained in yield of 74.3%. $^1$H NMR (400 MHz, $CDCl_3$), $\delta$ (ppm): 9.04 (s, 2H), 7.64 (d, $J$ = 4.0 Hz, 8H), 7.44–7.40 (m, 12H). $^{13}$C NMR (100 MHz, $CDCl_3$), $\delta$ (ppm): 155.3, 140.4, 138.7, 130.0, 129.4, 128.8, 128.4. HRMS (MALDI-TOF): m/z 486.1920 ($[M]^+$), calcd for 486.1844). Crystal structure (CCDC 1822244).

**Synthesis of TAA-4TPA:** The synthetic procedure is similar to that of TAA-4P except that the benzil was replaced by 1,2-bis(4-(diphenylamino)phenyl)ethane-1,2-dione (**5**). The crude product was purified on a silica-gel column using hexane/ dichloromethane (v/v = 1:1) as eluent. A red powder of 320 mg (0.28 mmol) was obtained in yield of 40%. $^1$H NMR (400 MHz, $CD_2Cl_2$), $\delta$ (ppm): 8.94 (s, 2H), 7.61 (d, $J$ = 8.0 Hz, 8H), 7.38–7.34 (m, 16H), 7.21–7.14 (m, 24H), 7.08 (d, $J$ = 8.0 Hz, 8H). $^{13}$C NMR (100 MHz, $CDCl_3$), $\delta$ (ppm): 154.0, 148.4, 146.6, 139.7, 131.5, 130.3, 128.8, 127.2, 124.6, 123.0, 121.0. HRMS (MALDI-TOF): m/z 1154.4731 ($[M]^+$), calcd for 1154.4784).

**Synthesis of TAA-4PTPA:** The synthetic procedure is similar to that of TAA-4P except that the benzil was replaced by 1,2-bis(4'-(diphenylamino)-[1,1'-biphenyl]-4-yl)

ethane -1,2-dione. The crude product was purified by recrystallization in toluene. A red powder of 75 mg (0.05 mmol) was obtained in yield of 38.5%. $^1$H NMR (400 MHz, *d*-THF), $\delta$ (ppm): 8.94 (s, 2H), 7.83 (d, *J* = 8.0 Hz, 8H), 7.73 (d, *J* = 8.0 Hz, 8H), 7.67 (d, *J* = 8.0 Hz, 8H), 7.31 (t, *J* = 8.0 Hz, 16H), 7.17 (t, *J* = 8.0 Hz, 24H), 7.07 (t, *J* = 8.0 Hz, 8H). HRMS (MALDI-TOF): m/z 1458.6014 ([M]$^+$), calcd for 1458.6036).

**Fabrication of nanoparticles:** TAA-4PTPA NPs were fabricated from the aqueous self-assembly of TAA-4PTPA and amphiphilic DSPE-PEG$_{2000}$. Typically, TAA-4PTPA (2 mg) and DSPE-PEG$_{2000}$ (10 mg) were dissolved in 5 mL $CHCl_3$, then 45 mL deionized water was slowly added to the solution, then treated with ultrasonication and final evaporation to remove $CHCl_3$, affording the resultant stable TAA-4PTPA NPs.

**In Vitro Cytotoxicity Evaluation:** The cytotoxicity of TAA-4PTPA NPs was evaluated by MTT assay. HeLa cells were seeded in 96-well plates (100 μL) and incubated overnight at 37 $^o$C in a humidified 5% $CO_2$ atmosphere. The culture medium was refreshed and added with TAA-4PTPA NPs and incubated for 24 h. Then the medium in each well was replaced with fresh cell culture medium, and MTT reagent was added and incubated for another 6 h until purple precipitates were observed. Finally, DMSO (100 μL) was added to each well, and the plates were incubated at 37 $^o$C for 15 min. The absorbance intensity at 570 nm was recorded and the cytotoxicity was expressed as a percentage of the control.

**Cell imaging:** HeLa cells were cultured in DMEM with 10% fetal bovine serum (FBS), at 37 $^o$C in $CO_2$/air (5:95) in a humidified incubator. The fluorescence imaging was performed on a confocal laser scanning microscopy (CLSM). The late endosomes and lysosomes were stained with Lysotracker Green. For the photostability examination in cell imaging, HeLa cells were pretreated with TAA-4PTPA NPs for 3 h with dye concentration of 2 μM, then stained with Lysotracker Green and incubated for another 30 min. After that, the cell culture media was refreshed and washed with PBS for three times. The cells were continuously irradiated by the 488 nm laser on the CLSM imaging system, and the fluorescence images were taken at predetermined

irradiation time points: 0 min, 30 min and 60 min, respectively. These images were employed to evaluate the intracellular photostability of NPs and the commercial Lysotracker Green. The excitation wavelength was 488 nm, and the emission channel was set to be 500–560 nm and 600–730 nm for Lysotracker Green and NPs, respectively.

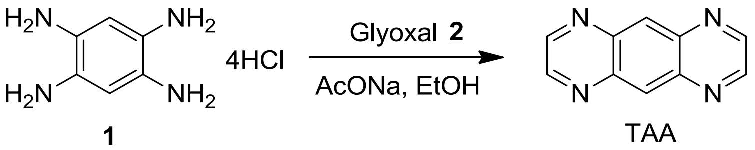


**Scheme S1.** Synthetic route to TAA.

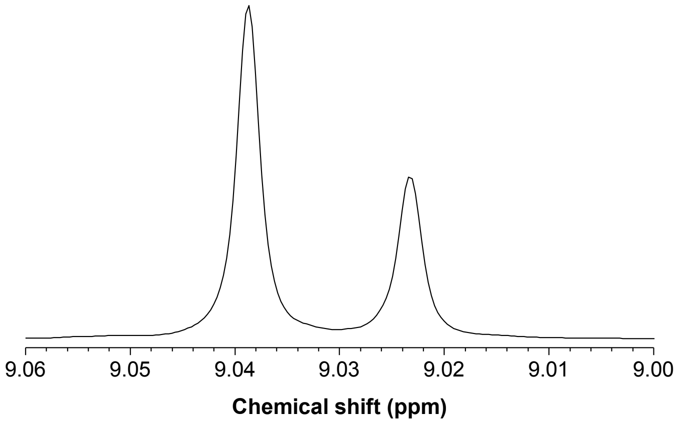


**Figure S1.** $^{1}$H NMR spectrum of TAA in $CDCl_3$.

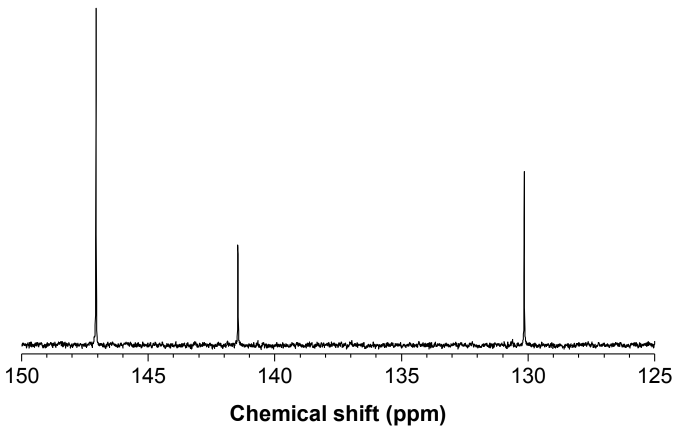


**Figure S2.** $^{13}$C NMR spectrum of TAA in $CDCl_3$.

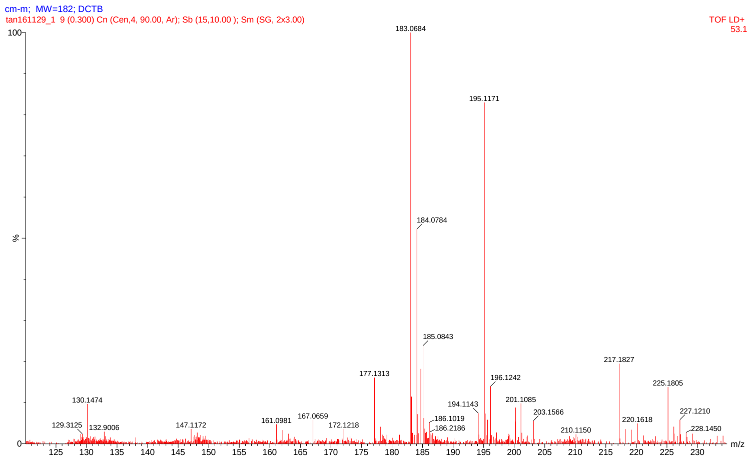


**Figure S3.** HRMS spectrum of TAA.

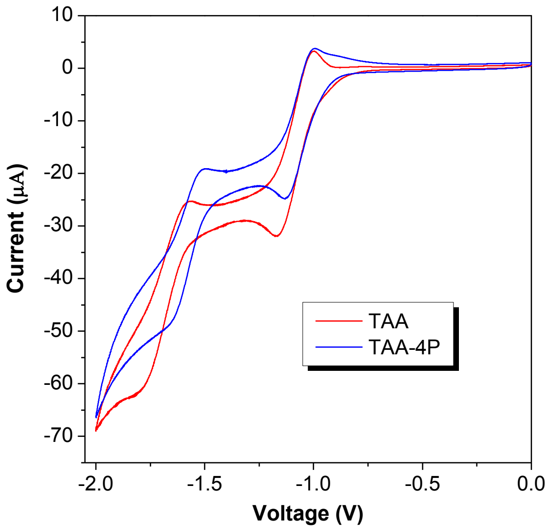


**Figure S4**. CV curves of TAA and TAA-4P in DCM at a scanning rate of 50 mV/s. $E_{\mathrm{LUMO}} = -(E^{\mathrm{red}}_{\mathrm{onset}} - E^{(1/2)}_{\mathrm{Fc/Fc+}} + 4.8)$ eV, $E^{(1/2)}_{\mathrm{Fc/Fc+}} = 0.47$eV.

The CV calculation showed that TAA showed a low LUMO energy level of -3.36 eV. Such value is much lower than that of commercial electron-transporting materials, such as tris(8-hydroxyquinolinato)aluminium (*ca.* -3.0 eV) and 1,3,5-tris (1-phenyl-1H-benzimidazol-2-yl)benzene (*ca.* -2.7 eV), indicating that TAA possessed high electron affinity.[5]

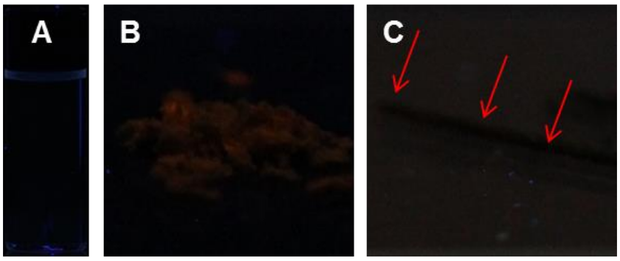


**Fugure S5.** Photographs of TAA in (A) THF, (B) pristine powder and (C) crystal taken under 365 nm UV light.

Nearly no emission can be observed under UV light and their spectra were hard to detect by fluorescence spectrometer.

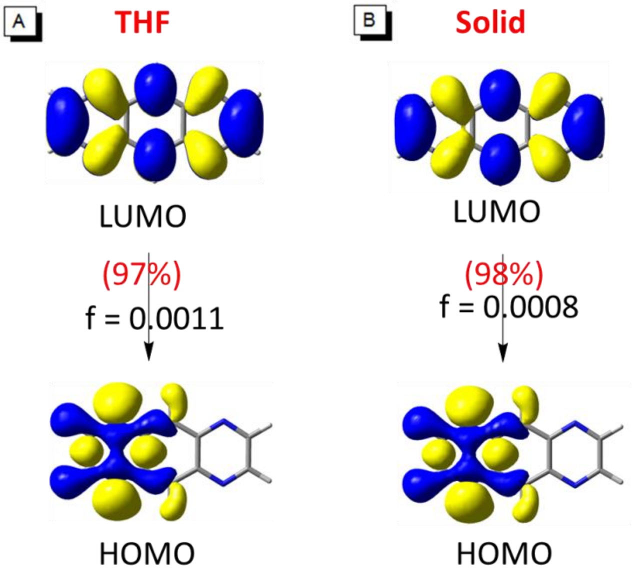


**Figure S6**. Conformation and transition information at the $S_1$min of TAA calculated at (TD) B3LYP/6-31G(d) level in (A) THF solution and (B) crystal state modeled by PCM and ONIOM (53 molecules) approaches.

Theoretical calculation revealed that a low oscillator strength (*f*) of *ca.* 0.001 from $S_1 \rightarrow S_0$ transition was obtained, while more than 97% of the component was contributed by the $n \rightarrow \pi^*$ transition.

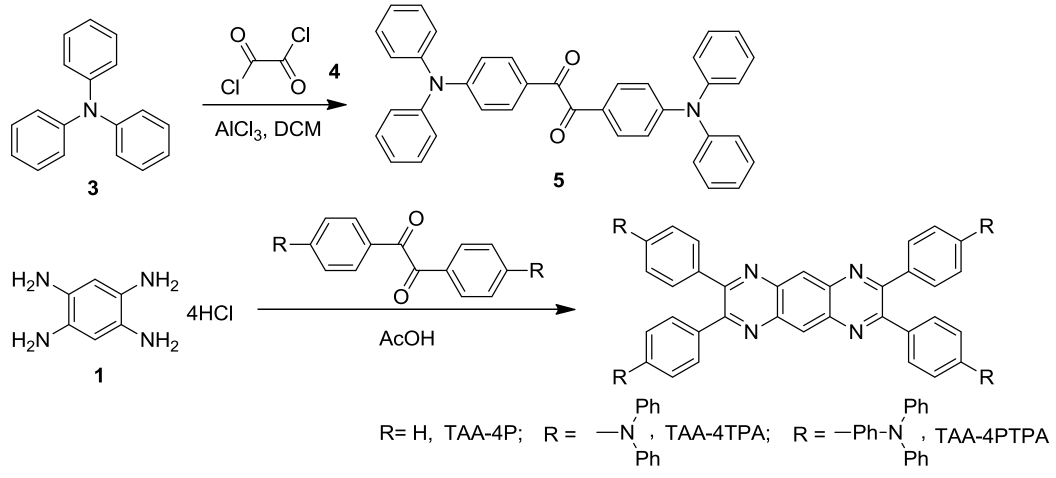


**Scheme S2.** Synthetic route to TAA-4P, TAA-4TPA and TAA-4PTPA.

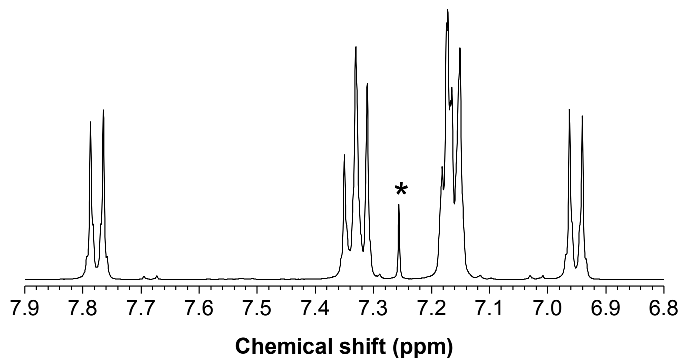


**Figure S7.** $^{1}$H NMR spectrum of **5** in $CDCl_3$.

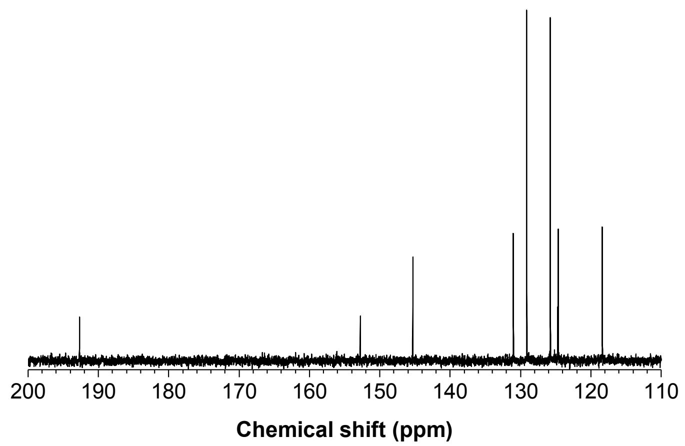


**Figure S8.** $^{13}$C NMR spectrum of **5** in $CDCl_3$.

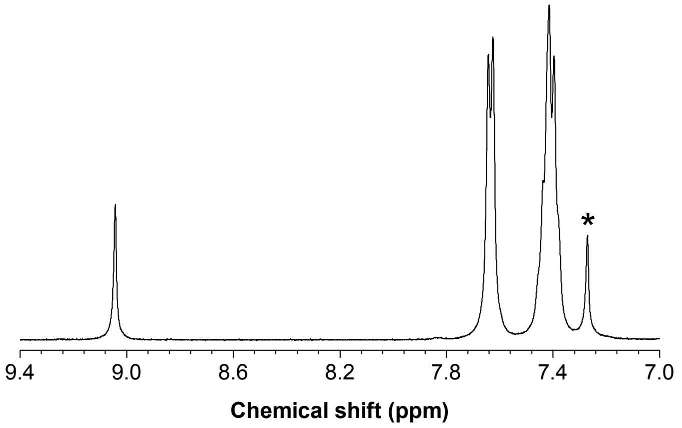


**Figure S9.** $^{1}$H NMR spectrum of TAA-4P in $CDCl_3$.

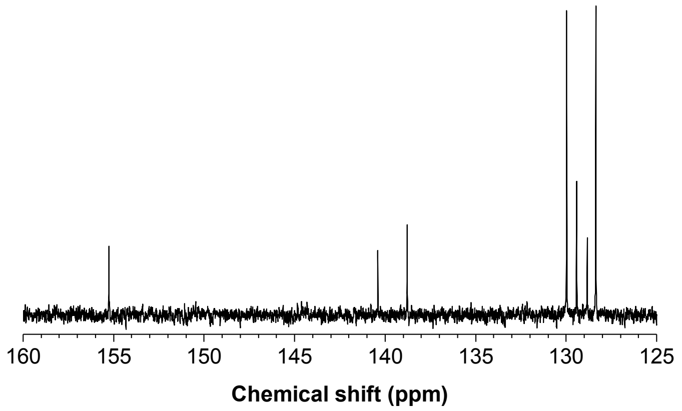


**Figure S10.** $^{13}$C NMR spectrum of TAA-4P in $CDCl_3$.

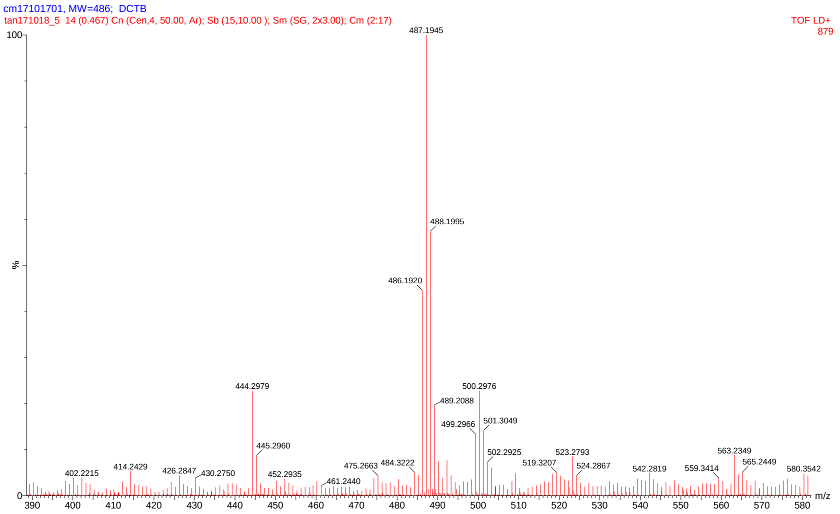


**Figure S11.** HRMS spectrum of TAA-4P.

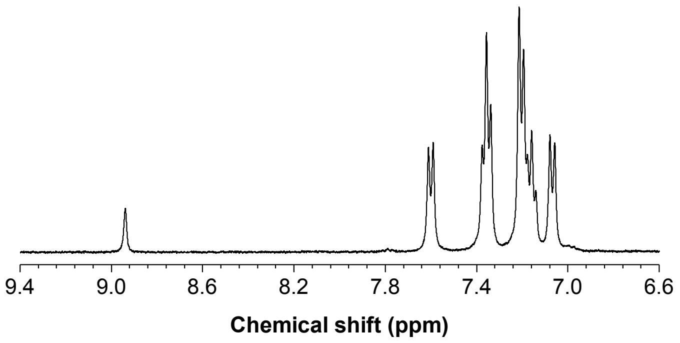


**Figure S12.** $^{1}H$ NMR spectrum of TAA-4TPA in $CD_2Cl_2$.

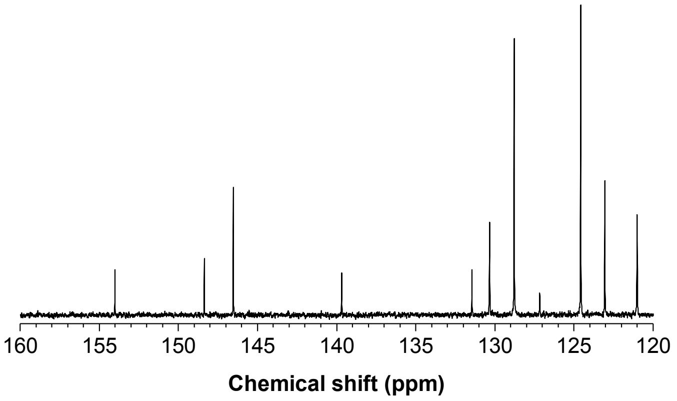


**Figure S13.** $^{13}C$ NMR spectrum of TAA-4TPA in $CDCl_3$.

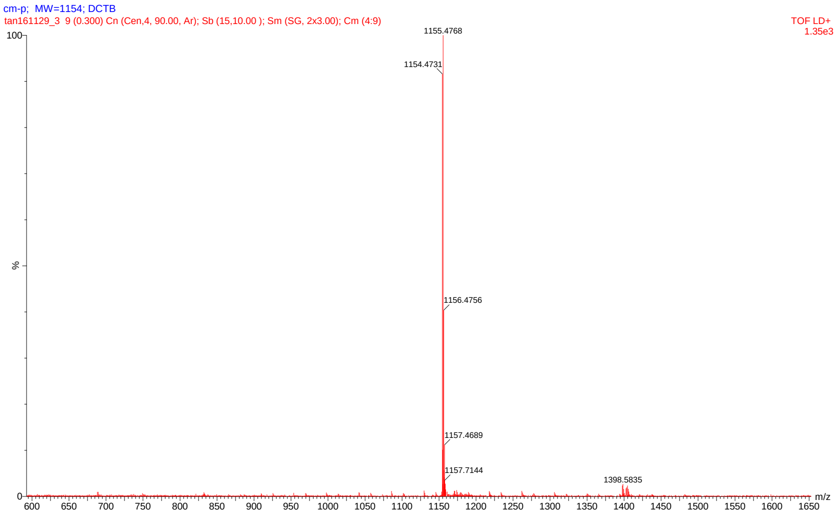


**Figure S14.** HRMS spectrum of TAA-4TPA.

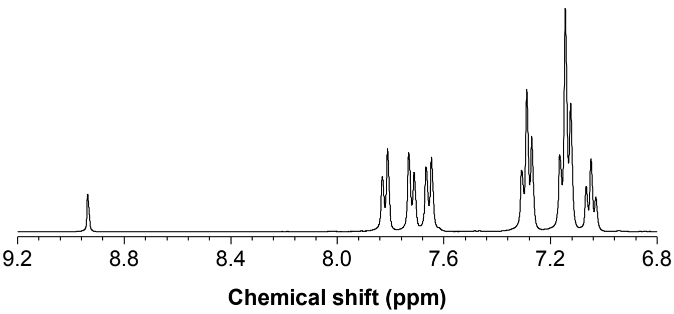


**Figure S15.** $^{1}$H NMR spectrum of TAA-4PTPA in *d*-THF.

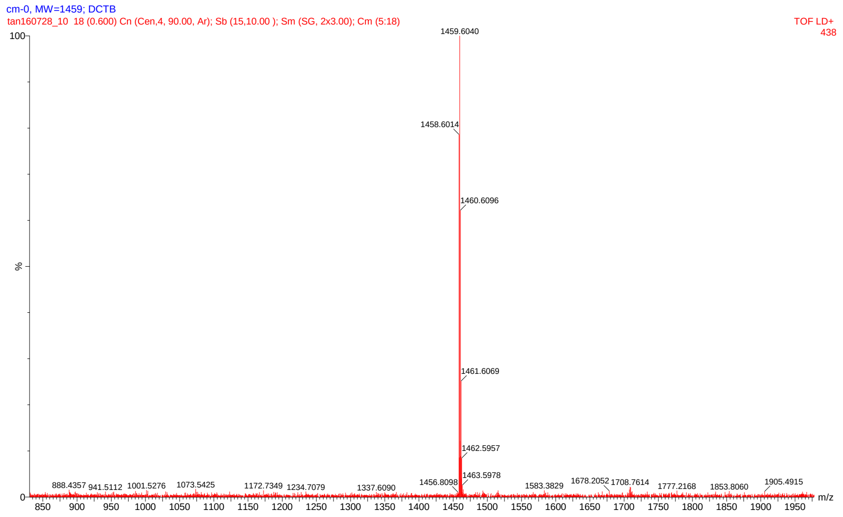


**Figure S16.** HRMS spectrum of TAA-4PTPA.

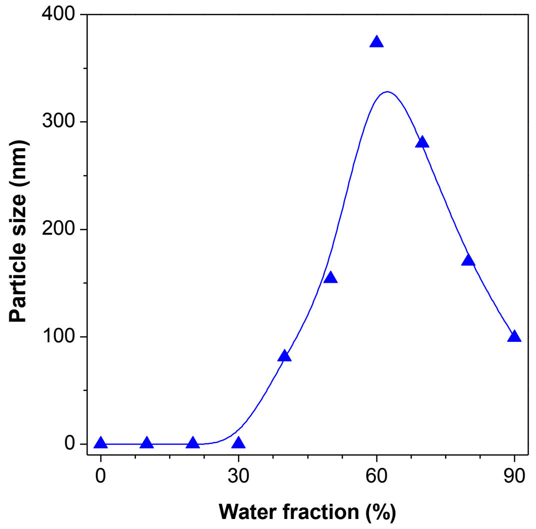


**Figure S17**. Particle sizes of TAA-4P in THF/water mixture with different *f*w.

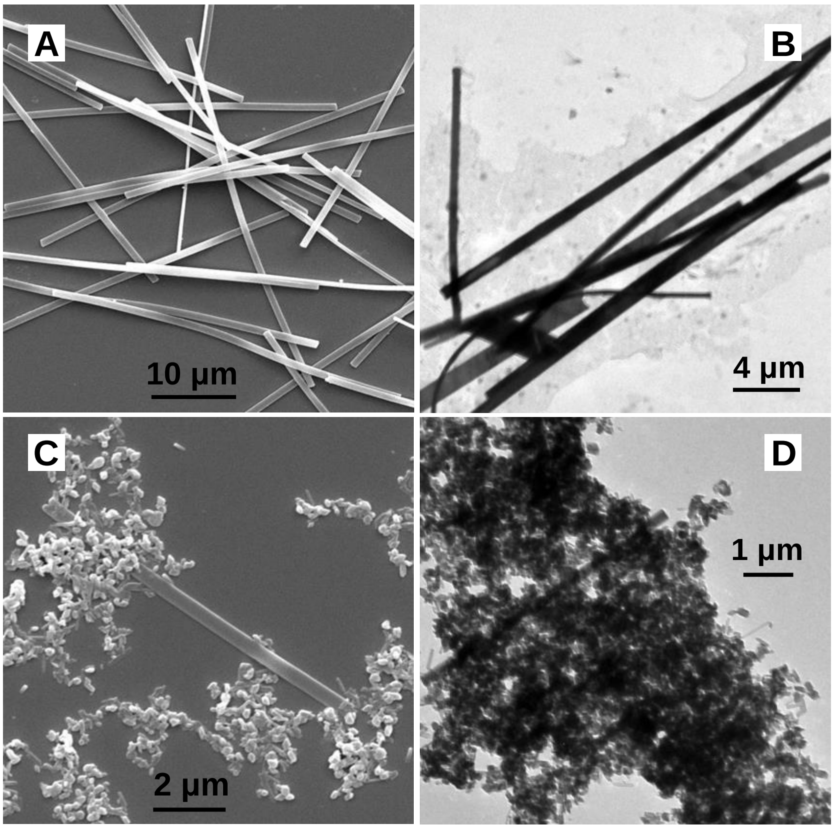


**Figure S18.** (A and C) SEM and (B and D) TEM images of assemblies of TAA-4P formed by nature evaporation of its THF/water mixtures with fw of (A and B) 70% and (C and D) 90%.

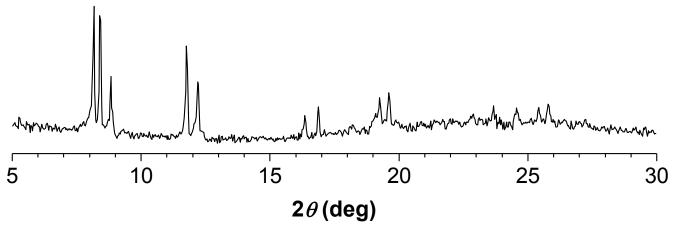


**Figure S19**. PXRD diffractogram of assemblies of TAA-4P formed after solvent was evaporated form THF/water mixture with *f*w = 70%.

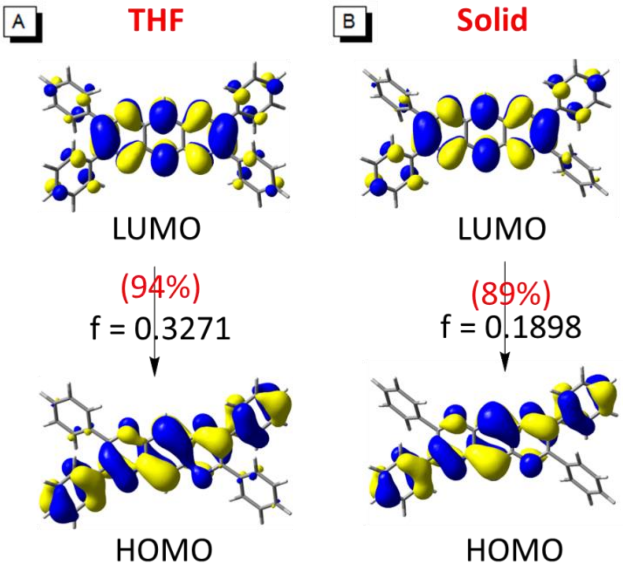


**Figure S20**. Conformation and transition information at the $S_1$min of TAA-4P calculated at (TD) B3LYP/6-31G(d) level in (A) THF solution and (B) crystal state modeled by PCM and ONIOM (43 molecules) approaches.

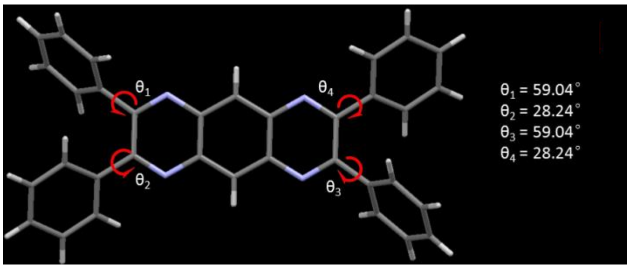


**Figure S21**. Molecular conformation of TAA-4P in the crystal.

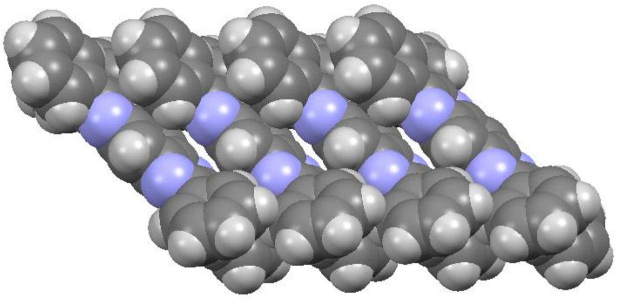

**Figure S22.** Molecular packing of TAA-4P in the crystal.

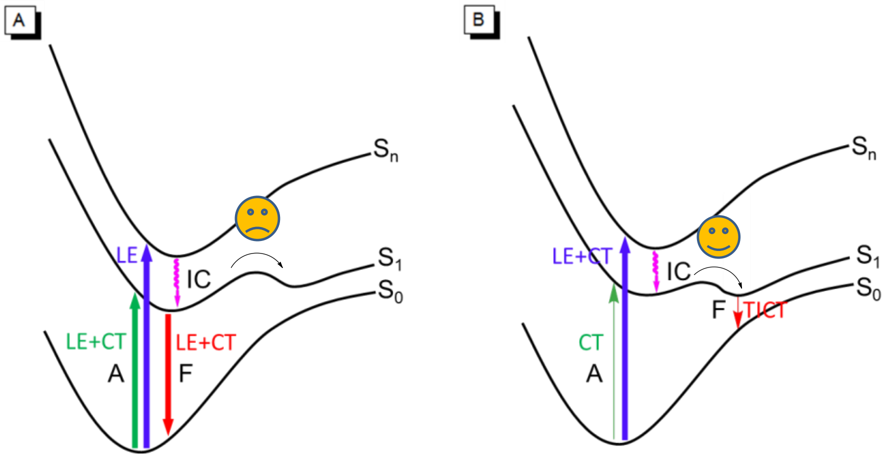


**Figure S23**. Proposed emission mechanisms of (A) TAA-4TPA and (B) TAA-4PTPA in the solution.

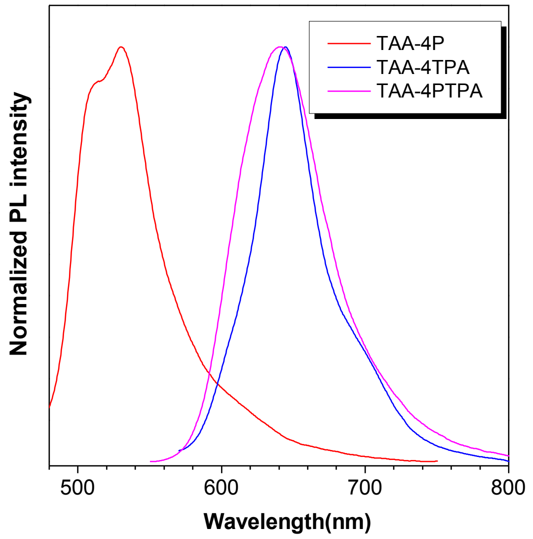


**Figure S24**. PL Spectra of TAA-4P ($\lambda_{ex}$ = 428 nm), TAA-4TPA ($\lambda_{ex}$ = 520 nm) and TAA-4PTPA ($\lambda_{ex}$ = 474 nm) in the solid powders.

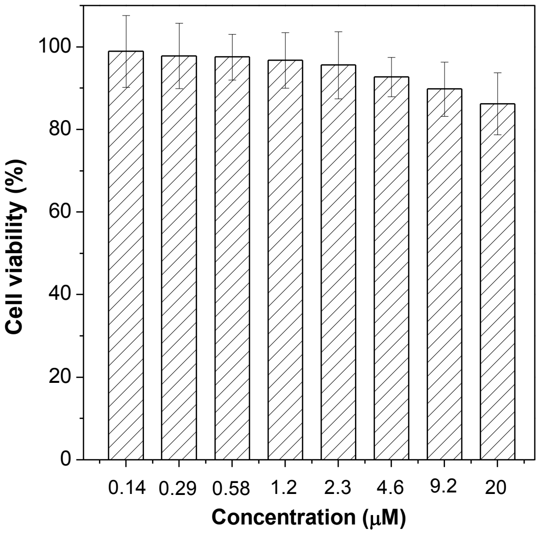


**Figure S25**. Cell viability of HeLa cells incubated with TAA-4TPA NPs for 24 h at different dye concentrations.

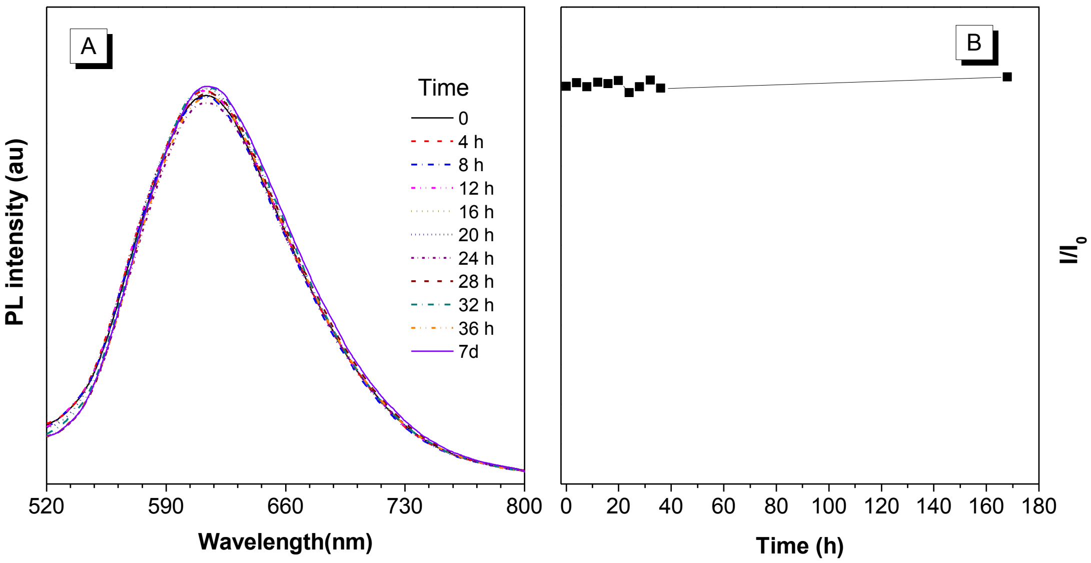


**Figure S26**. (A) PL spectra of NPs in water at pH = 5 as time increased, dye concentration: *ca.* 10 μM, $\lambda_{ex}$ = 474 nm. (B) Plot of relative PL intensity ($I/I_0$) versus time, where $I$ and $I_0$ are the PL intensity before and after treating with acid.

**Table S1.** Photo-physical properties of TAA-4P, TAA-4TPA and TAA-4PTPA

| | $\lambda_{abs}$ (nm) | $\lambda_{em,soln}$ (nm) | $\lambda_{em,solid}$ (nm) | Φ (%) | | $\tau$ (ns) | | $k_r$ (×10$^8$ s$^{-1}$) | | $k_{nr}$ (×10$^8$ s$^{-1}$) | |
|---|---|---|---|---|---|---|---|---|---|---|---|
| | | | | soln | solid | soln | solid | soln | solid | soln | solid |
| TAA-4P | 428 | 458,478 | 513,530 | 1.9 | 3.5 | 0.32 | 1.69 | 0.59 | 0.21 | 30.7 | 5.71 |
| TAA-4TPA | 520 | 627 | 645 | 19.7 | 4.9 | 3.22 | 2.07 | 0.61 | 0.24 | 2.49 | 4.59 |
| TAA-4PTPA | 474 | 708 | 640 | 1.0 | 8.0 | 1.59 | 6.72 | 0.06 | 0.12 | 6.23 | 1.37 |

$\tau$: average fluorescence lifetime calculated by $\tau = \sum A_i\tau_i^2/\sum A_i\tau_i$, where $A_i$ is the pre-exponential for lifetime $\tau_i$. Φ = fluorescence quantum yield measured by using an integrating sphere. $k_r$ = radiative decay rate (Φ/$\tau$). $k_{nr}$ = nonradiative decay rate (1/$\tau$ − $k_r$).

**Table S2.** Comparison of experimental data with results calculated by B3LYP and CAM-B3LYP

| | Absorption (nm) | | | Emission (nm) | | |
|---|---|---|---|---|---|---|
| | Exp. | B3LYP | Cam | Exp. | B3LYP | Cam |
| TAA-4P | 428 | 440 | 372 | 458,478 | 512 | 441 |
| TAA-4TPA | 520 | 636 | 438 | 627 | 723 | 467 |
| TAA-4PTPA | 474 | 634 | 406 | 708 | 731 | 434 |

Exp.: experimental results tested by UV-vis absorption and photoluminescence. B3LYP: results calculated by (TD) B3LYP/6-31G(d) in the PCM (Solvent=Tetrahydrofuran) model. Cam: results calculated by (TD) Cam-B3LYP/6-31G(d) in the PCM (Solvent=Tetrahydrofuran) model.

**Table S3.** Molecular orbitals and contribution of one-electron transition to excited state in absorption calculated by (TD) B3LYP/6-31G(d) in the PCM (Solvent=Tetrahydrofuran) model.

| | Electronic Configuration | Transition Orbitals | |
|---|---|---|---|
| TAA-4P | $S_0 \rightarrow S_1$ $\lambda$=440 nm<br>$f$=0.8890<br>HOMO→LUMO<br>97% | 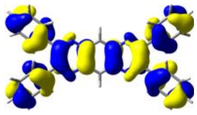<br>HOMO -6.15 eV | 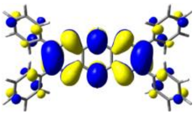<br>LUMO -2.80 eV |
| TAA-4TPA | $S_0 \rightarrow S_1$ $\lambda$=636 nm<br>$f$=1.0110<br>HOMO→LUMO<br>99% | 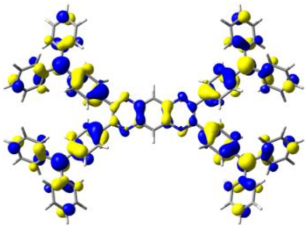<br>HOMO -4.98 eV | 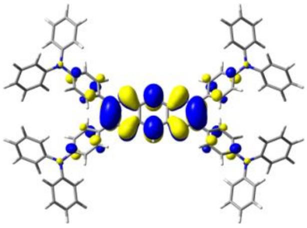<br>LUMO -2.66 eV |
| | $S_0 \rightarrow S_9$ $\lambda$=385 nm<br>$f$=1.0977<br>HOMO-2→LUMO+1<br>93% | 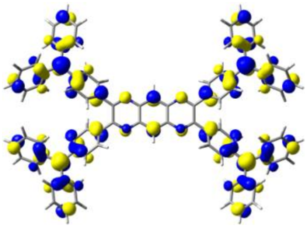<br>HOMO-2 -5.17 eV | 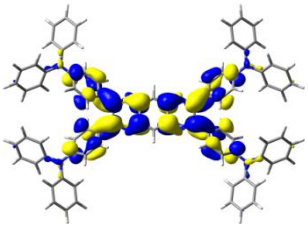<br>LUMO+1 -1.53 eV |
| TAA-4PTPA | $S_0 \rightarrow S_1$ $\lambda$=634 nm<br>$f$=0.6341<br>HOMO→LUMO<br>98% | 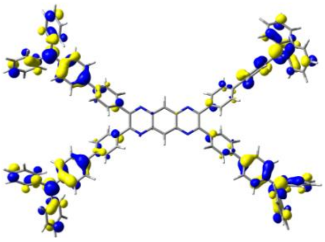<br>HOMO -5.01 eV | 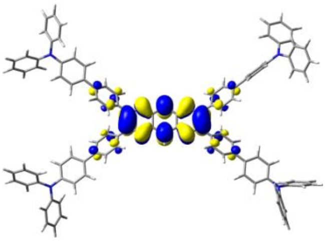<br>LUMO -2.78 eV |
| | $S_0 \rightarrow S_5$ $\lambda$=444 nm<br>$f$=1.1624<br>HOMO-4→LUMO<br>94% | 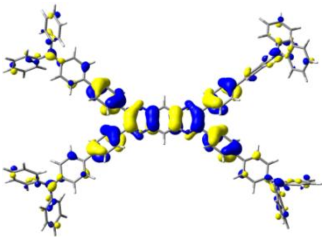<br>HOMO-4 -6.00 eV | 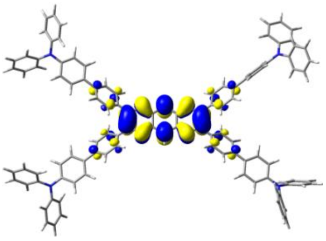<br>LUMO -2.78 eV |

**Table S4.** Molecular orbitals and transition information in emission calculated by (TD) B3LYP/6-31G(d) in the PCM (Solvent=Tetrahydrofuran) model.

| | Electronic Configuration | Transition Orbitals | |
|---|---|---|---|
| TAA-4 P | $S_1 \rightarrow S_0$ $\lambda$=512 nm<br>*f*=0.3271<br>LUMO→HOMO<br>94% | 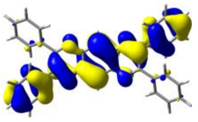<br>HOMO -5.87 eV | 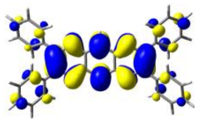<br>LUMO -3.05 eV |
| TAA-4 TPA | $S_1 \rightarrow S_0$ $\lambda$=723 nm<br>*f*=0.8175<br>LUMO→HOMO<br>99% | 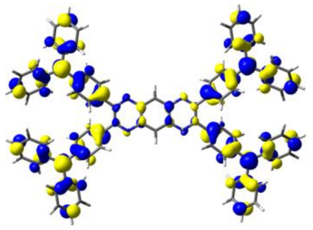<br>HOMO -4.99 eV | 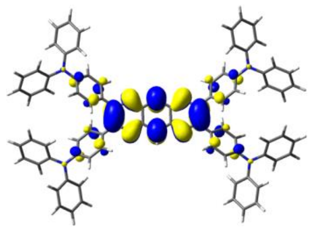<br>LUMO -2.93 eV |
| TAA-4 PTPA | $S_1 \rightarrow S_0$ $\lambda$=731 nm<br>*f*=0.3060<br>LUMO→HOMO<br>97% | 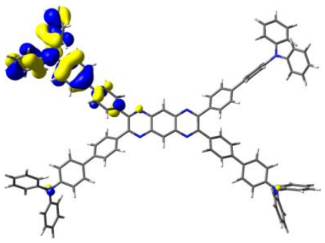<br>HOMO -4.96 eV | 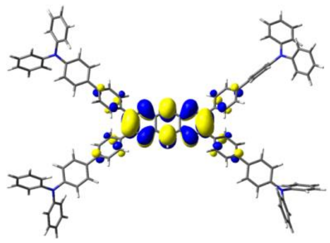<br>LUMO -3.01 eV |

## References


1. Y. Wang, M. Chen, N. Alifu, S. Li, W. Qin, A. Qin, B. Z. Tang, J. Qian, *ACS nano*, 2017, **11**, 10452.
2. T. Kobayashi, S. Kobayashi, *Eur. J. Org. Chem.*, 2002, 2066.
3. C. Wang, B. Hu, J. Wang, J. Gao, G. Li, W. W. Xiong, B. Zou, M. Suzuki, N. Aratani, H. Yamada, F. Huo, P. S. Lee, Q. Zhang, *Chem. Asian J.*, 2015, **10**, 116.
4. a) H. Takeshita, A. Mori, T. Nagao, T. Nagamura, *Chem. Lett.*, 1989, **18**, 1719; b) P. K. Sahoo, C. Ciri, T. S. Haldar, R. Puttreddy, K. Rissanen, P. Mal, *Eur. J. Org. Chem.*, 2016, **7**, 1283.
5. a) F. M. Hsu, C. H. Chien, P. I. Shih, C. F. Shu, *Chem. Mater.*, 2009, **21**, 1017; b) M. E. Kondakova, T. D. Pawlik, R. H. Young, D. J. Giesen, D. Y. Kondakov, C. T. Brown, J. C. Deaton, J. R. Lenhard, K. P. Klubek, *J. Appl. Phys.*, 2008, **104**, 094501.
